# A combined study of pharmacodynamics and pharmacokinetics of methamphetamine and its metabolite in male mice

**DOI:** 10.1186/s40001-025-03686-x

**Published:** 2025-12-23

**Authors:** Hongliang Su, Lingxiao Wang, Xiaoxiao Huang, Jinkai Wang, Yi Zhang, Yanhua Li, Yan Du, Xiaomeng Sun, Chao Zhang, Zhiwen Wei, Keming Yun

**Affiliations:** 1 School of Forensic Medicine, Shanxi Medical University, Taiyuan, 030001 PR China; 2 Key Laboratory of Forensic Toxicology, Ministry of Public Security, Beijing, 100192 PR China; 3 Department of Foreign Languages, Shanxi Medical University, Taiyuan, 030001 PR China; 4 School of Pharmaceutical Science, Shanxi Medical University, Taiyuan, 030001 PR China; 5 Technical Appraisal Research Center of the People’s Procuratorate of Shanxi Province, Taiyuan, 030021 PR China

**Keywords:** Methamphetamine, Pharmacodynamics, Pharmacokinetic, Locomotor activity, Mice

## Abstract

**Background:**

Methamphetamine (METH) is typically characterized as a potent dependent psychostimulant. Various behavioral models have been used to study METH drug dependence. The objective of this study was to determine the time period during which the neuropharmacological effects of METH are most pronounced, through a combined study of pharmacodynamics and pharmacokinetics.

**Methods:**

The present study simultaneously investigated the pharmacodynamics and pharmacokinetics of METH and its metabolite amphetamine (AMP) in mice by measuring the locomotor activity level and the concentrations of METH and AMP in the blood and brain from 5 min to 8 h after administration.

**Results:**

The pharmacodynamic analysis showed that the mice maintained a high level of activity from 5 min to 1 h after the administration, followed by a significant decrease in activity at 2 h. The pharmacokinetic analysis showed that METH was rapidly absorbed into the blood, and its concentration reached the peak value at 5 min. AMP concentration in blood increased and reached the peak value at 1 h; the concentrations of METH and AMP in the brain both reached the peak value at 30 min.

**Conclusions:**

In terms of pharmacodynamics and pharmacokinetics, the behavioral effects of METH on mice are most obvious within 1 h, at most 2 h, after administration, which can thus be considered a preferred time period to study the neuropharmacology effects of METH.

**Supplementary Information:**

The online version contains supplementary material available at 10.1186/s40001-025-03686-x.

## Introduction

Substance use disorder is deemed a chronic and recurrent brain disease caused by the continuous effect of dependent drugs on the brain, and it is mainly manifested in forced medication and persistent desire for drugs regardless of consequences, that is, people with substance use disorder lose control of drug craving and intake [1–4]. As an analogue of amphetamine (AMP), methamphetamine (METH) is typically characterized as a potent and dependent psychostimulant [5]. According to *The World Drug Report 2021* [6], there were 27 million users of AMP type stimulants in 2019 alone, accounting for about 10 percent of all drug abusers.

Pharmacological studies demonstrate that METH induces rapid central nervous system stimulation within 5 min of administration by enhancing dopamine (DA) release in synaptic spaces, triggering intense euphoria and progressive hyperactivity [7–12]. This acute neurochemical effect coincides with hepatic biotransformation through cytochrome P450 metabolism, where CYP2D mediates two parallel detoxification pathways: (1) aromatic hydroxylation producing 4-hydroxymethamphetamine (4-OH-METH), and (2) N-demethylation generating pharmacologically active amphetamine [13, 14].

Previous research showed that the female mice maintained the maximal levels of locomotor activity for 30 min–60 min after subcutaneous METH administration [15]. Although one study reported that intravenous METH (1.0 mg/kg) induced a significant increase in rat locomotion, lasting up to 3 h [16], the precise pharmacodynamic and pharmacokinetic profile in mice over an extended time course had not been fully characterized. Further pharmacokinetic analyses by Jaehne et al. revealed that METH and AMP levels in mice were higher at 30 min than 120 min after a 2 mg/kg dose [17]. To determine the time period during which the neuropharmacological effects are most pronounced after METH administration, we studied the spontaneous locomotor activity of mice and metabolic processes of METH and AMP in both blood and brain tissues. This experiment was designed to simultaneously investigate the pharmacodynamics and pharmacokinetics of METH and its metabolite AMP in mice during 5 min to 8 h after a single 2.0 mg/kg METH intraperitoneal (i.p.) administration. In this experiment, we first detected the locomotor activity level of mice by using spontaneous activity box and then determined the concentration of METH and AMP in blood and brain of mice by using high performance liquid chromatography-tandem mass spectrometry (HPLC–MS/MS).

## Materials and methods

### Animals

Male C57BL/6 mice (7 weeks old, 20–25 g) were obtained from the SPF (Beijing) Biotechnology Co., Ltd. (Beijing, PR China). They were housed in groups of four per cage under controlled temperature and humidity conditions, with a 12-h light/dark cycle (lights on at 07:00 and off at 19:00). Food and drinking water were provided ad libitum. All behavioral experiments were conducted during the daytime. All animal experiments complied with the ARRIVE guidelines were approved by Institutional Animal Care and Use Committee of Shanxi Medical University (approval Nos. 2021GLL051 [March 3, 2021]) and performed in accordance with the current relevant legislation in China.

### Drugs and reagents

*d-*Methamphetamine hydrochloride (purchased from the National Institutes for Food and Drug Control, NIFDC, Beijing, PR China) is dissolved in sterile normal saline (NaCl 0.9%). Amphetamine and D5-methamphetamine (D5-METH) standards are purchased from Cerilliant (Beijing, PR China). The volume of intraperitoneal injection was 10.0 ml/kg. The dosage of METH used in this experiment was 2.0 mg/kg, and the concentration of the administered METH was 0.2 mg/mL, which induced obvious behavioral sensitization in our previous studies [18].

### The pharmacodynamics study of METH in mice

The locomotor activity test was performed in an open field box (40 cm × 40 cm × 40 cm) with a silver grid bottom plate and an infrared camera on the top to track the movement of animals. The intelligent video tracking system records the autonomous activities of mice, and the Smart Small Animal Behavior Recording and Analysis System analyzes the trajectory (JLBeHv, PR China). The software can analyze the spontaneous activities of the mice as a whole or analyze their trajectories in any period.

Before the locomotor activity experiment, all mice were kept in the animal housing room for 1 week. Experimenters approached cages to handle the mice on a daily basis. First let the mice smell a stationary hand, and then gently stroke the mice on the neck and back when they feel comfortable. The experimenters then briefly pick up the mice using a cupping method, with handling duration gradually increased over several days. This protocol was implemented to minimize stress on the mice and reduce interference from experimental stimulation. Sixty-four mice were randomly divided into 7 METH subgroups (*n* = 8) and saline control group (*n* = 8). METH (2.0 mg/kg) or corresponding volume of normal saline was injected i.p. once. After administration, the mice were immediately placed into the locomotor activity boxes for experimentation until they were humanely euthanized 8 h later. During this period, an intelligent video tracking system continuously recorded the movement tracks of the mice, and then the tracks within 5 min were calculated and analyzed, starting from 5 min before the following time points of 5 min, 15 min, 30 min, 1 h, 2 h, 4 h, and 8 h, respectively. All mice in each METH subgroup were humanely euthanized instantly by cervical dislocation for the determination of METH and AMP concentrations in blood and brain after the movement track was collected.

### The pharmacokinetic studies of METH and its metabolite in mice

#### Sample preparation and HPLC–MS/MS analysis

After collecting movement trajectories, the mice were euthanized via cervical dislocation. Following ligation of the pulmonary artery, aorta, and precaval and postcaval veins, heart blood, and brain tissue were rapidly harvested, immediately processed, and analyzed via HPLC–MS/MS to determine METH and AMP concentrations in mice. 50 mg brain taken from the homogenized whole brain of an individual mouse or 50 μL blood was added into 1.5 mL centrifuge tube; then the following operations were carried out successively: added 10 μL internal standard (IS) working solution (D5-METH, 50 ng/mL), then added 600 μL acetonitrile methanol mixed solution (3:1), vortexed for 1 min, centrifuged (15,000 rpm/min, 4 °C) for 15 min, took the supernatant and evaporated to dryness at 40 °C water bath under a nitrogen stream (20–25 Psi), and reconstituted in 200 μL initial mobile phase and prepared for sample injection.

HPLC–MS/MS was used to determine the concentrations of METH and AMP in the brain and blood of mice. Chromatographic conditions: Agilent Zorbax Eclipse plus-c18 column (2.1 × 100 mm, 3.5 μm), column temperature: 25 °C, mobile phase: phase A was 0.1% formic acid, phase B was methanol, injection volume: 5 μl. Flow rate: 0.4 ml/min. The gradient elution condition was 0 ~ 1 min, 5% B; 1 ~ 2 min, 15% B; 2 ~ 4 min, 30% B; 4 ~ 5 min, 5% B. The injection volume was 5 μL. The column temperature was at 25 °C. The ionization mode was Electron Spray Ionization (ESI). The Multiple Reaction Monitoring (MRM) mode was used. The parameters of target compounds are listed in Supplementary Table 1.

#### Method validation

Validation of the assay was assessed according to FDA guidelines [19]. A total of seven calibrators were used to evaluate linearity in blood and brain by plotting the peak area ratio of METH or AMP to the IS versus the concentrations of METH or AMP. The seven calibrators of METH and AMP in blood were 1, 10, 50, 100, 200, 500, and 1000 ng/mL and 0.1, 1, 5, 10, 20, 50, and 100 ng/mL, respectively. The seven calibrators of METH and AMP in brain were 1, 10, 50, 100, 200, 500, and 1000 ng/g.

The limit of detection (LOD) and the lower limit of quantification (LLOQ) were determined by extracting dilutions of a low calibrator using blood and brain from six different lots of mice. The six calibrators of METH and AMP in blood were 0.05, 0.1, 0.2, 0.5, 0.8, and 1 ng/mL and 0.01, 0.05, 0.1, 0.2, 0.5, and 0.8 ng/mL, respectively. The six calibrators of both METH and AMP in brain were 0.1, 0.5, 1, 5, 10, and 50 ng/g. LOD is defined as the lowest concentration of analyte that still gave a signal-to-noise ratio (S/N) of 3. LLOQ is defined as the lowest concentration of analyte that repeatedly gave an S/N of 10 with a relative error of the mean (REM, in %) of < 20%. Accuracy and precision were evaluated at four quality control (QC) concentration levels of METH and AMP: in blood, LLOQ (1 ng/mL and 0.1 ng/mL), low (10 ng/mL and 1 ng/mL), medium (100 ng/mL and 10 ng/mL), and high (1000 ng/mL and 100 ng/mL) in six replicates (n = 6); in brain these concentration levels include LLOQ (1 ng/g), low (10 ng/g), medium (50 ng/g), and high (1000 ng/g). Accuracy is represented by relative error (RE, in %), and precision is represented by Coefficient of Variation (CV, in %).

The recoveries, matrix effect, and stability of METH and AMP from blood or brain during the extraction process were determined after the analyte concentrations from samples spiked before extraction were compared at three levels (low, medium, and high) with those from samples spiked after extraction (*n* = 8).

### Data statistical analysis

All data were represented by mean ± *SEM*, unless otherwise stated, and analyzed by SPSS (IBM SPSS Statistics for Windows, IBM Corp., USA, Version 26.0). Two-way ANOVA was used to analyze the changes of activity of mice at different time points after METH administration, followed by Tukey multiple comparisons post hoc test which was conducted to analyze the activity amount differences of the same group at different time points and different groups at the same time point. The dynamic distribution of METH and its main metabolite AMP in brain and blood was analyzed with one-way ANOVA, followed by Tukey multiple comparisons post hoc test which was conducted to analyze the concentration differences at different time points. The population pharmacokinetic model analysis of METH and AMP in mice was performed using a non-compartmental model (NCM) with Phoenix WinNonlin software (Pharsight Corporation, Mountain View, CA, Version 8.1). t_1/2_ is the elimination half-life; AUC_0-t_ is the area under the concentration–time curve; CL/F is the clearance; Vd/F is the distribution volume; T_max_ is the time point at which the drug concentration reaches its maximum.* t* test was used to compare the pharmacokinetic parameters of METH and AMP. The *p* values less than 0.05 are considered statistically significant.

## Results

### Method validation

No interference was visually observed at the retention time of analytes in blank blood and brain. The chromatograms of METH and AMP for the calibrator of LOD in blood and brain of mice are shown in Supplementary Fig. 1. The parameters of LOD, LLOQ, accuracy, precision, recovery, matrix effect, and stability are described in Supplementary Table 2, which met requirements specified in FDA guidelines.

The pharmacodynamics of METH in mice.

**Fig. 1:**
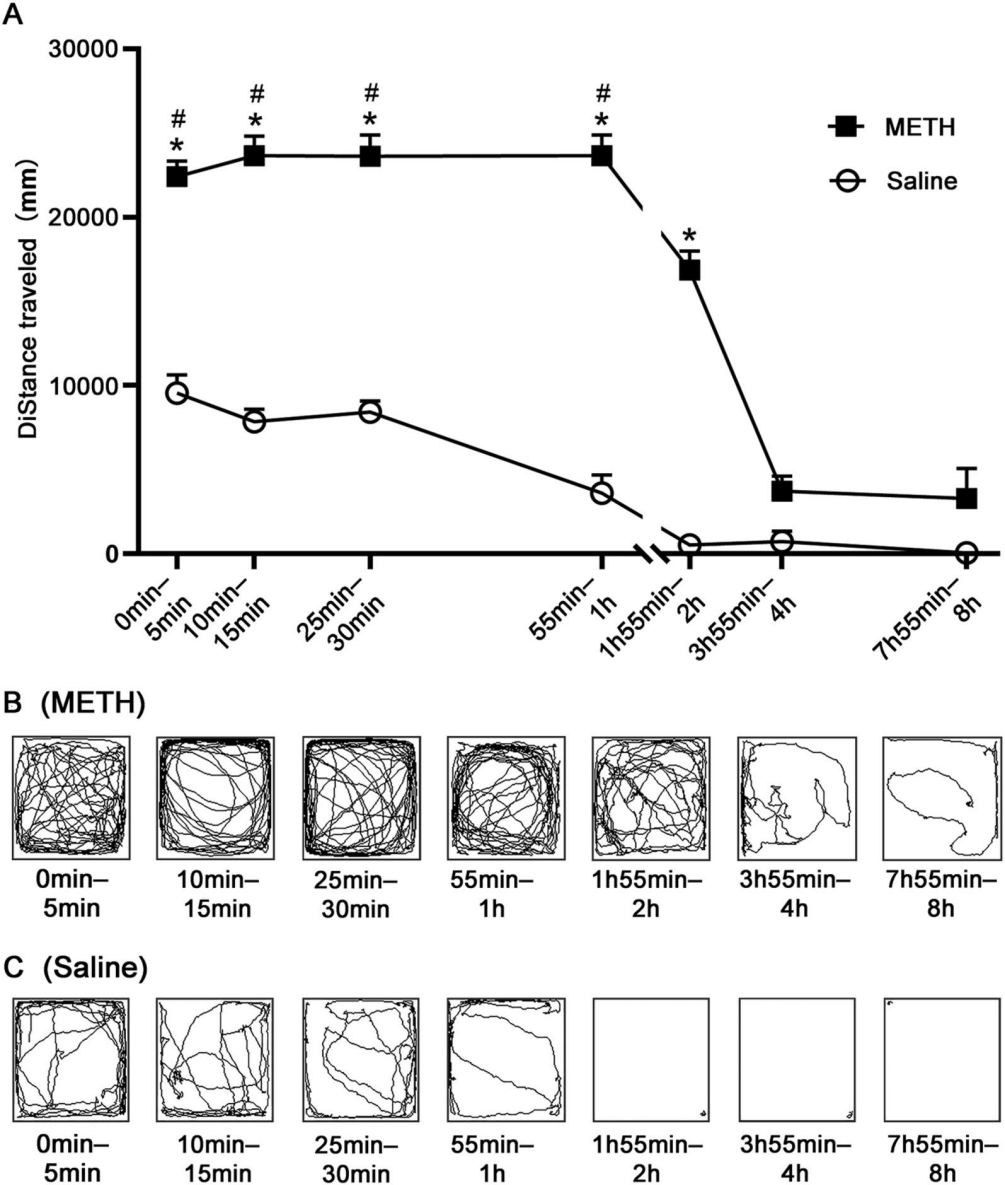
The changes of activity of mice at different time. **A** the activity amounts within 5 min of mice at time points of 5 min, 15 min, 30 min, 1 h, 2 h, 4 h, and 8 h (starting from 5 min before the time points) after i.p. 2.0 mg/kg METH or saline administration. The movement track of a representative mouse after methamphetamine (**B**) or saline (**C**) administration. Data are represented by mean ± SEM. ^*^p < 0.05 compared with the saline group at the same time point, ^#^*p* < 0.05 compared with the METH group at 2 h

The changes of activity of mice at different time points under the action of METH (2.0 mg/kg) are shown in Fig. 1. The results of two-way ANOVA showed that both drug [F_drug_(1, 246) = 164.233, *p* < 0.001] and time [F_time_(6, 246) = 23.259, *p* < 0.001] had significant effects, and the interaction between them was significant [F_drug*time_(6, 246) = 6.051, *p* < 0.001].

We compared the activity amount of mice in METH group with that of mice in saline group at all time points and found that the activity amount of mice in METH group was significantly higher at 5 min, 15 min, 30 min, 1 h, and 2 h, compared with the saline group (*p* < 0.05, Fig. 1A), whereas there was no significant difference at 4 h and 8 h (*p* > 0.05, Fig. 1A). Afterward, we compared the activity amount of mice at different time points and found that: in the METH group, the mice maintained a high level of activity amount from 5 min to 1 h (compared with 2 h, *p* < 0.05, Fig. 1A), which began to decline at 2 h, and then the mice maintained a low level from 4 to 8 h; in the saline group, the mice entered a fresh environment and explored actively at first, and then their spontaneous activities gradually decreased over time (Fig. 1A).

An intelligent video tracking system was used to collect the movement tracks of the mice within 5 min of each time point. As shown in Fig. 1B, C, in the METH group, the mice repeatedly moved around the test box in the first 1 h, and the movement tracks in the middle area were fewer than those near the sidewalls of the box, exhibiting obvious characteristics of adherent movement. After that, the adherent movement of mice gradually disappeared and the movement tracks decreased (Fig. 1B); in the saline group, the movement tracks of mice in the first 1 h were in disorder and spread all over the test box, and then the movement of mice gradually decreased (Fig. 1C).

Taken together, these results suggested that the increase of activity amount in mice induced by METH (2.0 mg/kg) was time dependent, and the mice maintained a relatively high level within 2 h (in particular within 1 h) after METH administration. No stereotypy was observed in the whole locomotor activity.

### The pharmacokinetics of METH and its metabolite in mice

#### The pharmacokinetics of METH and its metabolite in blood

**Fig. 2:**
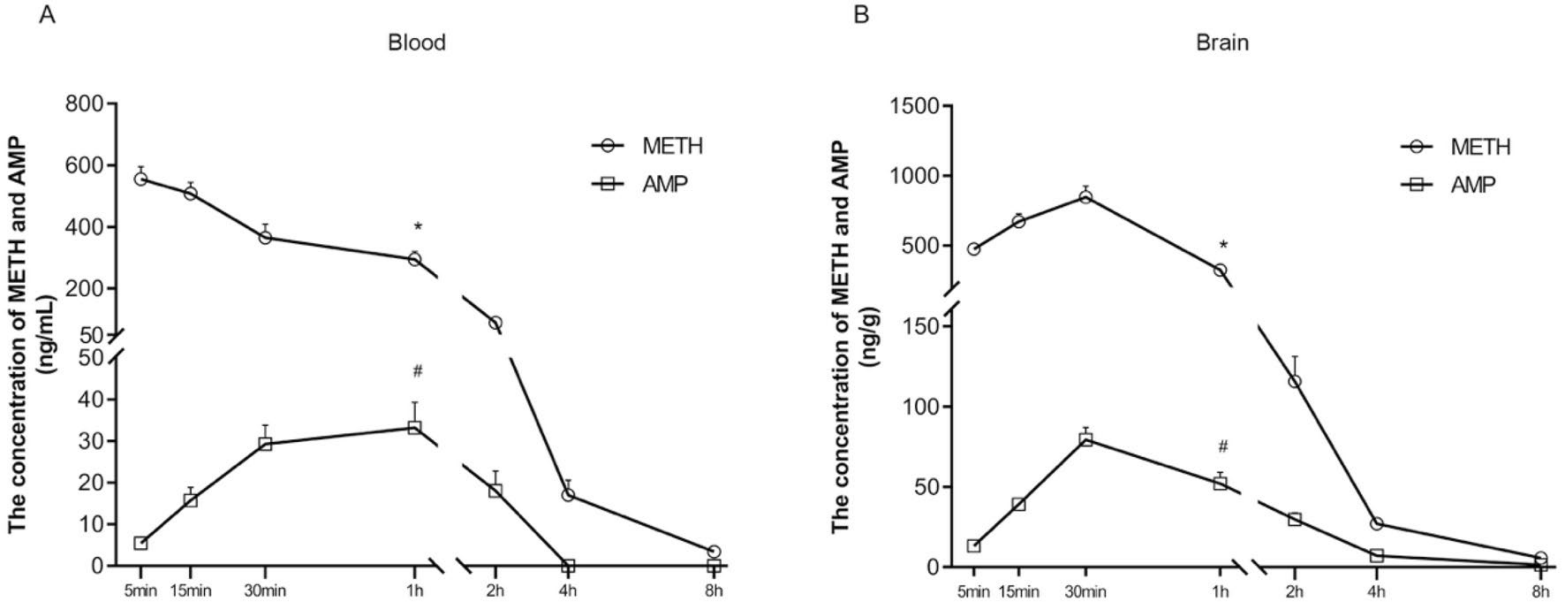
The concentrations of methamphetamine and amphetamine in the blood (**A**) and brain (**B**) of mice at 5 min, 15 min, 30 min, 1 h, 2 h, 4 h, and 8 h after i.p. 2.0 mg/kg METH administration. Data are represented by mean ± SEM. ^*^p < 0.05 compared with the concentration of METH at 2 h, ^#^*p* < 0.05 compared with the concentration of AMP at 2 h

After i.p. administration of METH (2 mg/kg), the concentrations of METH and AMP in blood of mice were monitored during 5 min to 8 h (Fig. 2A). The results of one-way ANOVA showed that time had significant effect on the distribution of METH [F_METH_ (6, 49) = 40.344, *p* < 0.001] and AMP [F_AMP_ (6, 49) = 14.554, *p* < 0.001]. METH was rapidly absorbed into the blood and at 5 min its concentration reached the peak value of 555.188 ± 40.432 ng/mL. Then its concentration decreased gradually over time, but it could still be detected up to 8 h later. AMP was detected at 5 min and reached its peak value of 33.211 ± 6.044 ng/mL at 1 h. Then its concentration decreased gradually over time, and the concentration fell below the detection limit at 4 h.

#### The pharmacokinetics of METH and its metabolite in brain

Similarly, time also had significant effects on the distribution of METH [F_METH_ (6, 49) = 59.664, *p* < 0.001] and AMP [F_AMP_ (6, 49) = 38.212, *p* < 0.001] in brain (Fig. 2B). METH and AMP were detected at 5 min and reached their peak values of 832.376 ± 70.012 ng/g and 79.176 ± 8.130 ng/g at 30 min, respectively. Then their concentrations decreased gradually over time, but they could still be detected up to 8 h later. The concentrations of METH and AMP remained at a relatively high level in the first hour and began to decrease significantly shortly thereafter (compared with 2 h, *p* < 0.05).

#### The pharmacokinetic parameters of METH and its metabolite in blood and brain

Using WinNonlin pharmacokinetic software, the average pharmacokinetic parameters of METH and AMP after a single i.p. 2.0 mg/kg METH administration in mice are summarized in Table 1. As to the different compounds in the same tissue, the elimination half-life, clearance, distribution volume, and time of maximum concentration of METH were lower than those of AMP in both blood and brain (*p* < 0.01, except the time of maximum concentration of METH in brain, *p* > 0.05), and the area under the concentration–time curve of METH in blood and brain was about 7–10 times that of AMP (*p* < 0.01). As to the same compound in different tissues, the clearance and distribution volume of METH and AMP in brain were higher than those in blood, and the time of maximum concentration of METH in brain was higher than that in blood.

**Table 1:** Pharmacokinetic parameters of METH and AMP in heart blood and brain of mice

Pharmacokinetic parameters	Heart blood		Brain	
	METH	AMP	METH	AMP
t_1/2_ (h)	0.58 ± 0.28*	1.10 ± 0.12	1.12 ± 0.36#	1.23 ± 0.36
AUC_0-t_ (μg/mL*h or ng/g*h)	714.57 ± 84.82*	59.25 ± 23.35	1144.84 ± 334.20*#	152.26 ± 24.11#
CL/F (L/h/kg)	2.83 ± 0.31*	39.45 ± 17.22	1.83 ± 0.35*#	13.27 ± 2.29#
Vd/F (L/kg)	2.40 ± 1.38*	22.42 ± 12.08	2.99 ± 1.26*	23.96 ± 9.79
T_max_ (h)	0.12 ± 0.08*	0.88 ± 0.52	0.44 ± 0.12#	0.56 ± 0.18#

t_1/2_, elimination half-life; AUC_0-t_, area under the concentration–time curve; CL/F, clearance; Vd/F, distribution volume; T_max_, time of maximum concentration

^*^p < 0.05 compared with those of amphetamine in the same tissue

^#^*p* < 0.05 compared with those of the same compound in heart blood

To sum up, pharmacokinetic results showed that the concentration of METH in blood decreased gradually over time, whereas the concentration of AMP in blood and the concentration of METH and AMP in brain first increased and then decreased over time. In view of the high concentrations of METH and AMP in both blood and brain within 1 h (or 2 h, to be cautious) after METH administration, we suggest that this time period be considered for studying the neuropharmacology effects of METH.

## Discussion

In humans, METH is mainly excreted through urine and metabolized in the liver by CYP2D6 hydroxylation and demethylation, converting it into 4-hydroxy METH and AMP [9, 20, 21–23]. Although it is generally accepted that METH is more potent and leads to more dependence than AMP, its analogue, both are psychostimulants [22]. Like METH, AMP acts directly on the central nervous system and plays a central excitatory role by increasing the synaptic concentration of monoamine neurotransmitters [24, 25].

Previous studies found that the exposure to METH first in early adolescence can significantly enhance the horizontal movement of mice, and the increase of movement induced by METH is closely related to the increase of DA in nucleus accumbens [26, 27]. Ohia-Nwoko et al. found that, after subcutaneous injection METH (4 mg/kg) administration, the locomotor activity of female mice was increased at 10 min and maintained at maximal levels from 30 to 60 min, but locomotor activity was only recorded for 90 min post-injection [15]. Gorman et al. reported that subcutaneous methamphetamine (3 mg/kg) rapidly elevated locomotor activity of male mice within 30 min. This hyperactive state persisted for about 40 min and subsided to baseline after 169 ± 20 min [28]. The present study found that mice maintain a high level of activity amount within 1 h after a single i.p. METH administration and their activity amount began to decline at 2 h, and then the mice maintained a low level of activity amount at 4 h and 8 h (Fig. 1A), which was roughly consistent with a previous study in which METH (≤ 5 mg/kg, i.p.) induces hyperlocomotion and the activity amount of animals returns to the baseline within 3–4 h after drug administration [29]. These studies suggested that METH can enhance the locomotor activity of mice in a time-dependent manner, and a relatively high level can be maintained within 2 h (in particular within 1 h) after METH administration. Our results, together with previous findings, indicate that under the current experimental conditions, a single i.p. dose of METH enhances locomotor activity in a time-dependent manner, with a relatively high level maintained for up to 2 h (in particular within 1 h). Being highly fat soluble, METH can easily cross the blood–brain barrier into the brain after entering the blood, and its effect lasts for a long time since it tends to be cleared slowly in brain tissue; the prolonged duration of action can be attributed, at least in part, to the high lipid solubility of METH, which allows it to cross the blood–brain barrier easily and may also contribute to its slow clearance from brain tissue [21, 30, 31]. A previous study found that 1.0 mg/kg METH resulted in a significant increase in exercise up to 3 h, which is roughly consistent with our experimental results [16]. Other dependent drugs, such as cocaine, AMP, and morphine, can also lead to the increase in locomotor activity of mice at low dose [32–34].

It was found that environments and administration methods would influence rodents’ 5–20 min exploration behavior, thus causing interference to the study of spontaneous activities of drug-dependent mice. For example, some scholars conducted a pre-test experiment of normal saline before the drug experiment to make the mice adapt to the experimental environment [35] or put the mice in the spontaneous activity box for 5–20 min before drug administration. Similarly, we found in this experiment that the mice in the normal saline control group explored actively in the new environment, and their spontaneous activities gradually decreased over time. In addition, it can be expected that the mice move less in the open field box after 4 h without food and water. Nonetheless, it was found that the locomotor activity of mice in METH group was higher than that in saline group 4 h later.

As METH and AMP are highly lipid soluble, they can quickly pass through the blood–brain barrier after entering the blood. The concentrations of METH and AMP in brain reached the peak value at 30 min and then decreased, resulting in high concentrations of METH and AMP in brain within 1 h (within 2 h, to be cautious). Previous studies also showed that the effects of METH in organs (including brain and heart) from both humans and mice reached their maximum within an hour [36]. Pharmacokinetic software analysis indicated that t_1/2(METH)_ and t_1/2(AMP)_ were 51.93 ± 21.68 min and 72.99 ± 7.69 min, respectively. Tuv et al. found that, after i.p. (3.75 mg/kg) administration of METH, the concentrations of METH in blood and brain peaked at 5 min and 20 min, respectively, and the t_1/2_ of METH was 36 min and 45 min, respectively [37], which was roughly consistent with our present results. Gorman et al. observed that after subcutaneous administration of 3 mg/kg methamphetamine in mice, the Cmax was reached at 30 min in both serum and brain, with half-lives of 53 min in serum and 52 min in the brain, respectively [29]. The brain data are consistent with our results, but the blood Tmax (0.12 h) and half-life (0.58 h) in our study are notably shorter than those reported in the literature. This discrepancy may be attributed to differences in the administration method [38]. We chose i.p. route over the methods of mimicking clinical or abuse scenarios (e.g., intravenous or inhalation) to minimize procedural trauma in mice, which could confound the measurements of spontaneous locomotor activity. The i.p. administration enables rapid systemic absorption of methamphetamine in animals while reducing stress-related artifacts in behavioral assessment. The above-mentioned studies were roughly consistent with our present results (Table 1), in which the t_1/2_ of METH in brain was longer than in blood. In the present study, although the Vd of METH and AMP in brain and blood had no statistical difference, the clearance in blood was faster than that in brain. Compared with the T_max_ in blood, the T_max_ of amphetamine in brain was earlier observed, and this was presumably caused by its high lipid solubility, which facilitates rapid distribution across the blood–brain barrier, coupled with a relatively slow elimination rate in the brain. A combination of these factors contributes to a prolonged retention time of amphetamine in brain tissue. In conclusion, the relatively slow clearance process of METH and AMP in brain probably results in long-lasting exposure of the brain to the sympathomimetic effects of METH [39], accounting for the high level of locomotor activity of mice until 4 h after METH administration.

In terms of pharmacodynamics and pharmacokinetics, our data demonstrate that the behavioral effects of METH are most pronounced within the first hour after administration. This suggests that the early phase (≤ 1 h) may be the optimal stage for investigating the acute neuropharmacological effects of the drug. The significant decrease in effects observed 2 h later indicates that the timing of experimental assessments is a critical factor. Researchers should carefully consider these pharmacokinetic and pharmacodynamic profiles when selecting time points for their specific experimental objectives. Although the present study provides a clear characterization of the pharmacokinetic and pharmacodynamic profile in male mice, we acknowledge that inclusion of female subjects would add a critical dimension to understanding potential sex differences. Given that our design precludes such examination, future research including both sexes will be valuable for verifying the generalizability of these findings and developing a more comprehensive model of methamphetamine’s effects. In brief, the pharmacokinetic and temporal response characteristics defined in this study provide a foundation for designing future investigations into the neuropharmacology of METH.

## Supplementary Information

Supplementary Material 1.

## Data Availability

The datasets generated and analysed during the current study are available from the corresponding author on reasonable request.

## Abbreviations

METH: Methamphetamine
AMP: Amphetamine
DA: Dopamine
4-OH-METH: 4-Hydroxymethamphetamine
i.p.: Intraperitoneal
HPLC–MS/MS: High-performance liquid chromatography-tandem mass spectrometry
D5-METH: D5-methamphetamine
IS Internal: Standard
ESI Electron: Spray Ionization
MRM Multiple: Reaction Monitoring
LOD: The limit of detection
LLOQ: The lower limit of quantification
S/N: Signal-to-noise ratio
REM: Relative error of the mean
RE: Relative error
CV: Coefficient of Variation
t_1/2_: Elimination half-life
AUC_0-t_: Area under the concentration–time curve
CL/F: Clearance
Vd/F: Distribution volume
T_max_: Time of maximum concentration
