# Supplementary material for: A combined study of pharmacodynamics and pharmacokinetics of methamphetamine and its metabolite in male mice
Source: Eur J Med Res. 2025 Dec 23;31:133. doi: 10.1186/s40001-025-03686-x (PMC12838478; doi:10.1186/s40001-025-03686-x)
Supplement: Supplementary file 1 — Supplementary Material 1. [file 40001_2025_3686_MOESM1_ESM.docx]

**Materials and Methods**

**Supplementary Table 1** MRM parameters of target compounds

| Compound | Precursor ion (m/z) | Fragmentor | Product ion (m/z) | Collision energy (eV) |
| --- | --- | --- | --- | --- |
| METH | 150 | 70 | 118.9 | 5 |
|  |  |  | 91* | 17 |
| AMP | 136.1 | 70 | 90.8* | 15 |
|  |  |  | 118.7 | 5 |
| D5-METH | 155 | 75 | 92.1* | 5 |
|  |  |  | 120.8 | 17 |

* Quantitative ion

**s**

**Results**

**Supplementary Fig. 1**



 Supplementary Fig. 1 The chromatograms of methamphetamine (METH) and amphetamine (AMP) for the calibrator of LOD in blood (A) and brain (B) of mice.

**Supplementary Table 2:** Liner, LOD, LLOQ, accuracy, precision, recovery, matrix effect, and stability of the method for METH and AMP in mice tissues assay

| Tissues |  | Liner | | LOD  (ng/mL or ng/g) | LLOQ  (ng/mL or ng/g) | concentration | accuracy  (RE %) | | precision  (CV %) | | recovery  (%) | matrix effect | stability  (%) | |
| --- | --- | --- | --- | --- | --- | --- | --- | --- | --- | --- | --- | --- | --- | --- |
|  |  | equation | *r* |  |  |  | Intra-day | Inter-day | Intra-day | Inter-day |  |  | freeze and thaw | on-instrument |
| blood | METH | Y=6.316e^-3^ x+1.055e^-1^ | 0.9909 | 0.1 | 1 | LLOQ | 85.2 ± 3.4 | 100.2 ± 5.8 | 7.3 ± 1.1 | 7.3 ± 2.2 |  |  |  |  |
|  |  |  |  |  |  | L | 87.5 ± 7.1 | 86.5 ± 6.4 | 3.1 ± 0.6 | 2.3 ± 0.6 | 90.2 ± 2.4 | 82.1 ± 5.2 | 6.2 ± 0.2 | 4.5 ± 0.5 |
|  |  |  |  |  |  | M | 93.3 ± 6.2 | 91.1 ± 5.6 | 4.7 ± 0.7 | 5.7 ± 0.8 | 95.3 ± 5.1 | 83.2 ± 8.4 | 2.8 ± 0.2 | 3.4 ± 0.6 |
|  |  |  |  |  |  | H | 97.5 ± 9.1 | 83.2 ± 4.4 | 5.8 ± 1.5 | 4.7 ± 1.2 | 102 ± 8.5 | 79.2 ± 7.1 | 3.5 ± 0.4 | 5.1 ± 0.4 |
|  | AMP | Y=6.045e^-3^ x+7.785e^-2^ | 0.9985 | 0.05 | 0.1 | LLOQ | 89.0 ± 6.0 | 82.2 ± 2.9 | 3.8 ± 0.1 | 5.4 ± 0.1 |  |  |  |  |
|  |  |  |  |  |  | L | 83.7 ± 7.2 | 87.2 ± 6.6 | 3.5 ± 1.8 | 5.3 ± 1.8 | 88.5 ± 7.3 | 80.1 ± 4.5 | 6.2 ± 0.2 | 3.0 ± 0.3 |
|  |  |  |  |  |  | M | 92.1 ± 6.6 | 93.0 ± 6.6 | 4.9 ± 0.4 | 5.8 ± 0.5 | 104.4 ± 3.6 | 91.9 ± 7.8 | 5.7 ± 0.3 | 2.5 ± 0.7 |
|  |  |  |  |  |  | H | 87.3 ± 4.5 | 93.1 ± 5.7 | 5.1 ± 0.1 | 2.1 ± 0.4 | 93.5 ± 3.2 | 82.7 ± 9.3 | 4.2 ± 0.1 | 3.6 ± 1.0 |
| brain | METH | Y=5.398e^4^ x–5.582e^2^ | 0.9920 | 0.5 | 1 | LLOQ | 82.3 ± 2.7 | 99.7 ± 3.2 | 10.9±0.1 | 18.4±0.8 |  |  |  |  |
|  |  |  |  |  |  | L | 91.3 ± 6.1 | 93.6 ± 3.3 | 14.3±0.7 | 13.3±2.1 | 86.5 ± 4.2 | 92.1 ± 3.1 | 4.2 ± 1.2 | 2.0 ± 0.7 |
|  |  |  |  |  |  | M | 87.3 ± 5.4 | 97.0 ± 3.9 | 12.2±0.5 | 13.5±0.1 | 94.4 ± 5.3 | 89.9 ± 6.2 | 7.7 ± 0.7 | 2.6 ± 1.2 |
|  |  |  |  |  |  | H | 99.1 ± 6.3 | 88.3 ± 5.5 | 10.5±3.1 | 11.4±0.5 | 103.5 ± 2.6 | 94.7 ±3.5 | 5.2 ± 0.2 | 6.6 ± 1.5 |
|  | AMP | Y=7.281e^4^ x+1.455e^2^ | 0.9996 | 0.5 | 1 | LLOQ | 104.2 ± 8.5 | 92.8 ± 4.5 | 17.9±3.3 | 15.2±1.1 |  |  |  |  |
|  |  |  |  |  |  | L | 92.4 ± 5.4 | 95.2± 3.1 | 8.7±0.5 | 12.4±2.1 | 91.5 ± 8.1 | 83.1 ± 5.5 | 3.1 ± 0.2 | 4.1 ± 0.2 |
|  |  |  |  |  |  | M | 100.2 ± 3.9 | 99.9 ± 8.2 | 9.4±3.1 | 11.3±1.6 | 88.4 ± 2.5 | 87.9 ± 7.4 | 5.2 ± 0.3 | 3.5 ± 1.7 |
|  |  |  |  |  |  | H | 95.4 ± 8.8 | 87.6 ± 4.3 | 7.3±2.0 | 5.3±3.2 | 99.5 ±4.3 | 91.7 ± 6.2 | 8.0 ± 0.1 | 5.4 ± 1.0 |

Abbreviations: METH, methamphetamine; AMP, amphetamine; LOD, limit of detection; LLOQ, lower limit of quantification; L, low; M, medium; H, high
